# CsMYB73 negatively regulates theanine accumulation mediated by *CsGGT2* and *CsGGT4* in tea shoots (*Camellia sinensis*)

**DOI:** 10.1093/hr/uhae012

**Published:** 2024-01-10

**Authors:** Manman Chang, Ying Sun, Kangzhi Fang, Maoyin Fu, Jingyu Ma, Yang Gao, Qi Chen, Linlin Liu, Zhaoliang Zhang, Xiaochun Wan, Jun Sun

**Affiliations:** State Key Laboratory of Tea Plant Biology and Utilization, Anhui Agricultural University, 130 West Changjiang Road, Hefei City 230036, Anhui Province, China; College of Life Sciences, Anhui Agricultural University, 130 West Changjiang Road, Hefei City 230036, Anhui Province, China; State Key Laboratory of Tea Plant Biology and Utilization, Anhui Agricultural University, 130 West Changjiang Road, Hefei City 230036, Anhui Province, China; State Key Laboratory of Tea Plant Biology and Utilization, Anhui Agricultural University, 130 West Changjiang Road, Hefei City 230036, Anhui Province, China; State Key Laboratory of Tea Plant Biology and Utilization, Anhui Agricultural University, 130 West Changjiang Road, Hefei City 230036, Anhui Province, China; State Key Laboratory of Tea Plant Biology and Utilization, Anhui Agricultural University, 130 West Changjiang Road, Hefei City 230036, Anhui Province, China; State Key Laboratory of Tea Plant Biology and Utilization, Anhui Agricultural University, 130 West Changjiang Road, Hefei City 230036, Anhui Province, China; State Key Laboratory of Tea Plant Biology and Utilization, Anhui Agricultural University, 130 West Changjiang Road, Hefei City 230036, Anhui Province, China; State Key Laboratory of Tea Plant Biology and Utilization, Anhui Agricultural University, 130 West Changjiang Road, Hefei City 230036, Anhui Province, China; State Key Laboratory of Tea Plant Biology and Utilization, Anhui Agricultural University, 130 West Changjiang Road, Hefei City 230036, Anhui Province, China; State Key Laboratory of Tea Plant Biology and Utilization, Anhui Agricultural University, 130 West Changjiang Road, Hefei City 230036, Anhui Province, China; State Key Laboratory of Tea Plant Biology and Utilization, Anhui Agricultural University, 130 West Changjiang Road, Hefei City 230036, Anhui Province, China; College of Horticulture, Anhui Agricultural University, 130 West Changjiang Road, Hefei City 230036, Anhui Province, China

## Abstract

Theanine metabolism is a necessary biological process during the planting and production of tea that determines tea quality. There is currently little knowledge about the transcriptional regulation of theanine metabolism in tea plants. In this study, we demonstrated that γ-glutamyl-transpeptidase CsGGT4, as a homologous protein of the theanine hydrolase CsGGT2, exhibited a higher theanine synthesis catalytic efficiency. Homology modeling and molecular docking showed that differential protein structures between CsGGT2 and CsGGT4 implied their different biological functions in tea plants. Theanine content correlated significantly with the expression of *CsGGT2*, *CsGGT4* and the transcription factor *CsMYB73* in tea shoots from different seasons. Additionally, CsMYB73 was confirmed to act as a nucleus-localized transcription factor (TF), directly interacts with the *CsGGT2* and *CsGGT4* promoters, serving as an activator of *CsGGT2* and a suppressor of *CsGGT4*. Consequently, this leads to a negative association with theanine accumulation in tea shoots. Furthermore, the continuous increase in *CsMYB73* produced a significantly increase in *CsGGT2* expression and inhibited *CsGGT4* expression. The present study reveals that the degradation of theanine has been observed to increase, concomitantly with the inhibition of theanine synthesis, resulting in a significant decline in the accumulation of theanine in tea shoots during the process of seasonal greening in ‘Huangkui’ leaves. This study contributes to the broader comprehension of the intricate transcriptional regulatory hierarchy that governs the metabolism of theanine in tea shoots, offering novel approaches for managing tea plantations and enhancing tea quality.

## Introduction

As an economically important crop, tea leaves (*Camellia sinensis*) are generally selected for making tea, with good economic value and health effects. As a unique nonproteinogenic amino acid, theanine (γ-glutamyl-L-ethylamide) is dynamically regulated by environmental conditions and developmental cues [1, 2]. Similar to glutamine, theanine acts as a form of nitrogen storage and transport and has vital physiological functions in plant growth [3–7]. It is believed that nitrogen balance in tea plants is influenced primarily by the synthesis and degradation of theanine [8, 9]. Generally, theanine is mainly synthesized in the roots, which is transported to young leaves and other tissues of tea plant for use [2, 8, 10]. During leaf maturation, theanine levels decrease progressively from young leaves to older leaves [11, 12]. It is well documented that theanine accumulation is strongly influenced by factors such as seasons, development, and environmental influences [13–15]. Molecular mechanisms responsible for theanine biosynthesis and degradation, however, are yet to be elucidated.

**Figure 1:**
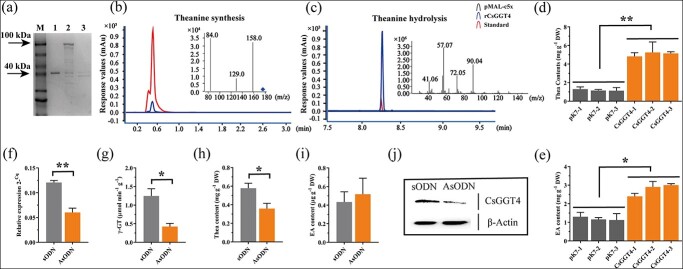
CsGGT4 has dual functions *in vitro* and in plant. **(a)** Analyses of recombinant protein rCsGGT4 by SDS–PAGE. Molecular mass protein standards are shown in Lane M with arrows indicating their size. Lanes 1, 2, and 3 represent the MBP protein, recombinant proteins rCsGGT2 and rCsGGT4, respectively. **(b)** Analysis of theanine synthase activity for recombinant protein rCsGGT4 by HPLC–MS/MS *in vitro*. **(c)** Analysis of theanine hydrolase activity for recombinant protein rCsGGT4 by GC–MS *in vitro*. **(d)** Theanine detection was conducted by HPLC–MS/MS on tobacco leaves overexpressing *CsGGT4* after injection of 10 mM ethylamine hydrochloride. **(e)** EA detection was conducted by GC–MS on tobacco leaves overexpressing *CsGGT4* after injection of 10 mM theanine. The leaves of ‘Shuchazao’ were injected with the AsODN-*CsGGT4* solution (50 μM), and sODN-*CsGGT4* supplied as a control. **(f)** Analysis of the *CsGGT4* expression levels in tea leaves with both control and silenced *CsGGT4*. **(g)** Total enzyme activity of γ-GT analysis in tea leaves with both control and silenced *CsGGT4*. **(h)** An analysis of theanine levels in tea leaves with both control and silenced *CsGGT4* by HPLC. **(i)** An analysis of ethylamine (EA) levels in tea leaves with both control and silenced *CsGGT4* by GC–MS. **(j)** Analysis of CsGGT4 protein expression levels in tea leaves with both control and silenced *CsGGT4* by western blot. A control protein β-actin derived from plants was used in this study. Based on data from three independent experiments. Statistics were conducted using the Student’s *t* test (^*^P < 0.05, ^**^P < 0.01, ^***^P < 0.001).

In tea plants, several core enzymes influence the metabolism of theanine. Theanine synthetase (CsTSΙ), glutamine synthetase (CsGS), γ-glutamyl-transpeptidase (CsGGT), and pyridoxine biosynthesis (CsPDX) are related to theanine synthesis and theanine degradation [16–18]*.* We previously demonstrated that CsGGT2 and CsGGT4 have both synthesis and degradation functions in the production of theanine *in vitro* and confirmed a light-regulated expression of *CsGGT2* in plants [19]. However, there is currently little research on how *CsGGT4* is regulated in tea plants.

Evidence has shown the significant involvement of transcription factors (TFs) in both biosynthesis and degradation processes of theanine. A genome-wide analysis of gene coexpression has revealed that the Myeloblastosis (MYB) transcription factor is responsible for regulating plant growth, stress response, hormone signaling, and metabolite biosynthesis [20, 21]. A high level of association between MYB transcription factors and theanine in tea plants has been confirmed [22]. CsMYB6 is specifically expressed in the tea roots and exerts its influence by binding to the promoter of *CsTSI*, thereby activating its expression [23]. Theanine biosynthesis is negatively regulated by CsMYB73 in tea plants, as shown by Wen *et al.* [24]. Despite the fact that plant nitrogen cycles are regulated by MYB transcription factors, few downstream genes have been extensively studied. Therefore, it is crucial to further investigate MYB TFs and their role in theanine metabolism.

Previously, we found that CsGGT2 functions as a photosensitive enzyme to degrade theanine in tea plants [19]. This study characterized the functions of CsGGT4 *in vitro* and *in planta*. As a result of their affinity for different substrates, CsGGT4 prefers theanine synthetase as demonstrated by enzyme kinetics analysis, transient expression in tobacco cells, and gene suppression in tea leaves. The homology modeling and molecular docking studies were conducted to explore the structural differences between CsGGT2 and CsGGT4 proteins and explore the possible reasons for their functional differences. Further experiments demonstrated that the transcription factor CsMYB73 bound directly to *CsGGT2* and *CsGGT4* promoters and negatively regulated theanine accumulation in the shoots of ‘Shuchazao’ from different seasons and in the shoots of ‘Huangkui’ during seasonal greening. Our findings offer a significant contribution to the understanding of theanine metabolism in tea plants, which could have important consequences for the enhancement of theanine levels in tea plant breeding.

**Figure 2:**
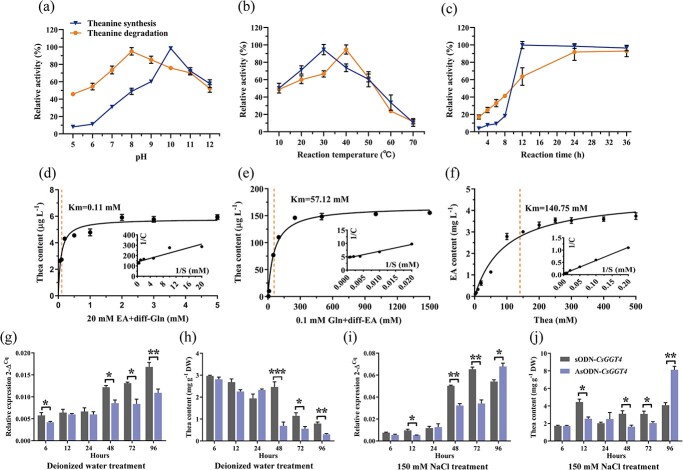
CsGGT4 has a preference for theanine synthetase *in vitro* and in tea plant. Enzyme activities were measured to determine the optimum pH **(a)**, temperature **(b)**, and reaction time **(c)** for rCsGGT4 protein. Theanine synthesis was initiated using 20 mM glutamine and 20 mM ethylamine hydrochloride, and degradation was initiated using 20 mM theanine. **(d)** Recombinant protein rCsGGT4 model for glutamine kinetics with the ethylamine hydrochloride (20 mM) was presented in reaction system. **(e)** Recombinant protein rCsGGT4 model for ethylamine hydrochloride kinetics with the glutamine (0.1 mM) was presented in reaction system. **(f)** Recombinant protein rCsGGT4 model for theanine kinetics. A solution of deionized water and 150 mM sodium chloride (NaCl) was applied to tea shoots for durations of 6, 12, 24, 48, 72, and 96 hours. **(g)** and **(h)***CsGGT4* expression levels and theanine contents in tea leaves with control and silenced *CsGGT4* after treatment with deionized water for various hours. **(i)** and **(j)***CsGGT4* expression levels and theanine contents in tea leaves with control and silenced *CsGGT4* after treatment with 150 mM NaCl for various hours. Assays were carried out on three independently conducted experiments and data were expressed as the mean ± SD (*n* = 3).

## Results

### Functional identification of CsGGT4, which synthesizes and degrades theanine *in vitro* and in tea plants

The previous study reported that CsGGT2 functions in tea leaves as a theanine hydrolase [19]. It is worth noting that there are two γ-glutamyl-transpeptidase coding proteins, CsGGT2 and CsGGT4, in tea plants [18]. To explore the biological function of CsGGT4 and verify its contribution to the theanine metabolism of tea plants, the enzyme activity of rCsGGT4 has been assessed *in vitro*. Unlike the theanine hydrolase CsGGT2, the purified rCsGGT4 protein is more prone to break and form two electrophoresis bands with an approximate size of 40 kDa, as observed in the SDS–PAGE analysis (Fig. 1a). The enzyme activity assays for the synthesis of theanine were conducted and analysed using HPLC–MS/MS under the specified preliminary conditions of 37°C for 24 h. Additionally, the degradation of theanine was analysed using GC–MS. According to the findings, the recombinant protein, rCsGGT4, successfully synthesized theanine from ethylamine hydrochloride and glutamine, as well as degrade it into ethylamine and glutamic acid *in vitro* (Fig. 1b and c).

Following ethylamine hydrochloride injection, tobacco leaves overexpressing *CsGGT4* exhibited significantly higher levels of theanine than those overexpressing the empty vector, and then theanine injection substantially increased ethylamine levels more than tobacco leaves overexpressing the empty vector (Fig. 1d and e). Based on these results, CsGGT4 from tea plant synthesizes and degrades theanine in tobacco cells. It is interesting to note that overexpressing CsGGT4 in tobacco leaves had a greater impact on theanine synthesis, demonstrating its functional differences from theanine hydrolase CsGGT2 [18].

AsODN-interfering antisense oligodeoxynucleotides (AsODN) was used to suppress the expression levels of *CsGGT4* in tea cuttings to investigate its function in theanine metabolism. As shown in Fig. 1f, tea leaves treated with AsODN-*CsGGT4* showed significant reductions in expression levels of *CsGGT4* compared to leaves treated with sense oligodeoxynucleotide sODN*-CsGGT4* (Fig. 1f). Upon treatment with AsODN, the protein levels of CsGGT4 and the total enzyme activity of γ-GT exhibited a significant decrease, as confirmed through western blotting and enzyme activity analysis. (Fig. 1g and j). In *CsGGT4*-silenced tea leaves, theanine levels were significantly decreased compared with the control leaves (sODN), whereas ethylamine (EA) levels were slightly increased (Fig. 1h and i). These results suggested that silencing *CsGGT4* would inhibit theanine synthetase activity in tea leaves. Moreover, there is evidence indicating that CsGGT4 may possess varying catalytic efficiency towards different substrates, with a higher propensity for theanine synthesis in tea plants.

### CsGGT4 has a preference for theanine synthetase in tea plants

A variety of thermodynamic parameters were measured for the synthesis and degradation of theanine by the recombinant protein rCsGGT4, including optimum pH, temperature, and reaction time. Theanine synthesis was best achieved by rCsGGT4 at pH 10 and 30°C after continuous reaction for 12 hours, just like the recombinant protein rCsGGT2. Additionally, after continuous reaction for 24 hours at pH 8 and 40°C, rCsGGT4 showed the best degradation efficiency of theanine (Fig. 2a–c). The present investigation involved the kinetic analysis of the recombinant protein rCsGGT4 to determine its affinity for substrates and catalytic efficiency. Results of kinetic analysis showed that rCsGGT4 had an estimated *km* value of 0.11 mM for glutamine, when 20 mM ethylamine hydrochloride was added in the reaction system (Fig. 2d), 57.12 mM for ethylamine hydrochloride, when 0.1 mM glutamine was added in the reaction system (Fig. 2e), and 140.75 mM for theanine (Fig. 2f). These results shown that CsGGT4 has lower *km* values for ethylamine and glutamine, indicating that CsGGT4 has stronger substrate affinity for ethylamine and glutamine than that for theanine. Preliminary demonstrated that CsGGT4 has higher synthesis efficiency of theanine. It was estimated that the equilibrium dissociation constants (*K_D_*) of rCsGGT4 for glutamine, ethylamine hydrochloride, and theanine were 0.039, 0.097, and 0.041 M, respectively (Fig. S1a–c, see online supplementary material). Based on these results, recombinant protein rCsGGT4 exhibits differential substrate affinity for various ligands *in vitro*. The affinity of rCsGGT4 to ethylamine hydrochloride (with 0.1 mM glutamine in reaction system) was significantly higher than that to theanine. Moreover, SPR analysis revealed a higher affinity of rCsGGT4 for ethylamine hydrochloride than CsGGT2 (Table 1).

**Table 1:** The results of molecular docking, enzyme kinetics, and SPR analysis for three ligands and proteins

Protein-Ligand	Number of H-bonds at binding site	Binding affinity (kcal/mol)	K_m_ (mM)	K_D_ (M)
CsGGT2-Theanine	3	−4.38	62.90 (ref. 19)	0.038 (ref. 19)
CsGGT4-Theanine	4	−4.14	140.75	0.041
CsGGT2-Glutamine	4	−4.71	0.04 (ref. 19)	0.024 (ref. 19)
CsGGT4-Glutamine	5	−5.09	0.11	0.039
CsGGT2-Ethylamine	2	−3.58	185.25 (ref. 19)	0.112 (ref. 19)
CsGGT4-Ethylamine	2	−4.45	57.12	0.097

Previous studies have suggested that salt stress induces theanine biosynthesize in tea plants [18]. Based on our previous research, we exposed tea shoots to deionized water or NaCl solution for various times to investigate the function of CsGGT4. A significant reduction in *CsGGT4* expression has been observed in tea leaves treated in deionized water with AsODN-*CsGGT4*, compared to control leaves treated with sODN-*CsGGT4* (Fig. 2g); while theanine was decreasing, the downward trend was greatly accelerated during the process (Fig. 2h). Figs 2i and 2j illustrate that when *CsGGT4* was expressed as significantly suppressed in tea leaves after 48 and 72 hours of salt treatment, a significant reduction in theanine was observed. Combined with the above results of the functional analysis of the recombinant protein rCsGGT4, we speculated that *CsGGT4* may be primarily responsible for theanine synthesis in tea plants.

### Homology modeling and structural analysis

In comparison with CsGGT2, CsGGT4 shared 48.25% amino acid identity, and possessed both a large and small subunit. Both have important structural motifs, including the catalytic residue threonine (Thr) located at the beginning of the small subunit, which has been identified in GGT from **Pseudomonas *aeruginosa* A-14 [25], as well as nucleophile residues glycine–glycine (Gly-Gly), proposed to be involved in stabilizing the enzymes tetrahedral transition state [26] (Fig. S2a, see online supplementary material). In addition, the secondary structure was predicted based on the amino acid sequences of CsGGT2 and CsGGT4. The results indicated that CsGGT4 has more helical structures than CsGGT2 (Fig. S2h and I, see online supplementary material).

In studies, it has been shown that proteins with a sequence identity higher than 25% have similar 3D structures [27, 28]. In this study, *Escherichia coli* GGT (E. ColiGGT, SMTL ID: 2e0w.1), which had undergone crystal analysis, was selected as the reference sequence. The identity of CsGGT2 and CsGGT4 with *E. coli* GGT was 35.54% and 35.22%, respectively. Therefore, it should be feasible to build 3D structures of CsGGT2 and CsGGT4 through homology modeling using *E. coli* GGT as the template. As shown in Fig. S2b–g, both CsGGT2 and CsGGT4 proteins are composed of two parts: large subunits and small subunits . Additionally, the 3D structure of CsGGT4 exhibits more compact and complex folding poses, which is consistent with the predicted results of the secondary structure (Fig. S2h and i). These results preliminarily reflected the differences in protein structure between CsGGT2 and CsGGT4.

### Binding modes generated by molecular docking

**Figure 3:**
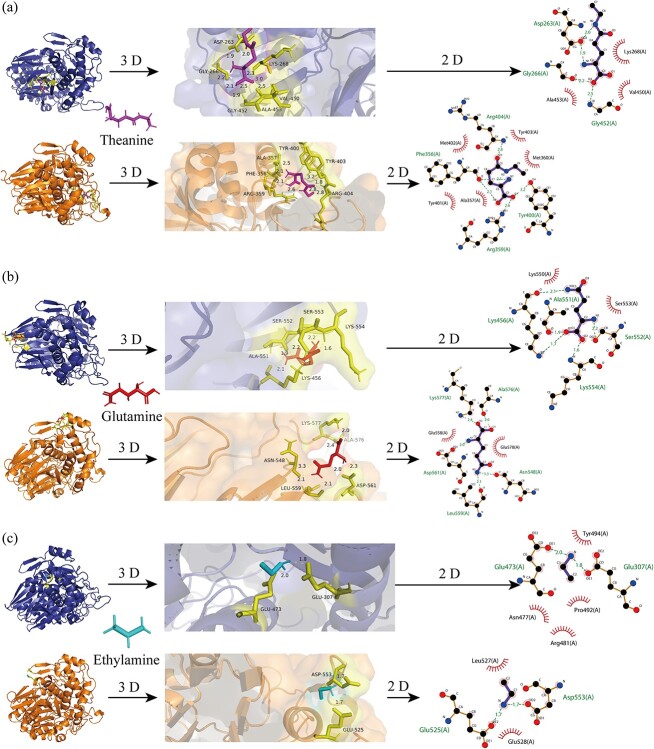
Molecular docking of CsGGT2 and CsGGT4 proteins. **(a)** Molecular docking of theanine binding to CsGGT2 and CsGGT4 proteins. **(b)** Molecular docking of glutamine binding to CsGGT2 and CsGGT4 proteins. **(c)** Molecular docking of ethylamine (EA) binding to CsGGT2 and CsGGT4 proteins. The CsGGT2 protein is displayed in blue, and the CsGGT4 protein is displayed in orange.

To identify the precise binding sites of theanine/glutamine/ethylamine in CsGGT2 and CsGGT4, molecular docking studies were carried out. The docking protocol was verified by redocking theanine, glutamine and ethylamine into the active sites of the CsGGT2 and CsGGT4 proteins. In molecular docking, binding energy indicates the binding efficiency of the ligand to the receptor and the likelihood of an effect. Smaller values indicate better ligand binding [29]. The binding poses for theanine, glutamine, and ethylamine on CsGGT2 and CsGGT4 are shown in Fig. 3. As seen in the image, the ligand and protein structure also displayed some noncovalent interactions. Docking analysis revealed that theanine and glutamine were most effectively bound on the surface of the CsGGT2 and CsGGT4 proteins (Fig. 3a and b). Interestingly, the binding cavity for ethylamine (EA) on the CsGGT2 protein was located inside the protein folding poses, which were considered to be unfavorable for interaction with the ligand. Instead, the binding cavity for ethylamine on the CsGGT4 protein was exposed on the protein surface (Fig. 3c). As reflected in Table 1, both CsGGT2 and CsGGT4 showed the best affinity to glutamine; CsGGT2 retained a higher binding ability to theanine than CsGGT4, while CsGGT4 persisted in a higher binding ability to EA than CsGGT2. Because EA has a more favorable binding affinity for CsGGT4, CsGGT4 has a higher efficiency in theanine synthesis than CsGGT2.

### *CsMYB73* is negatively correlated with theanine accumulation mediated by *CsGGT2* and *CsGGT4* in tea shoots

**Figure 4:**
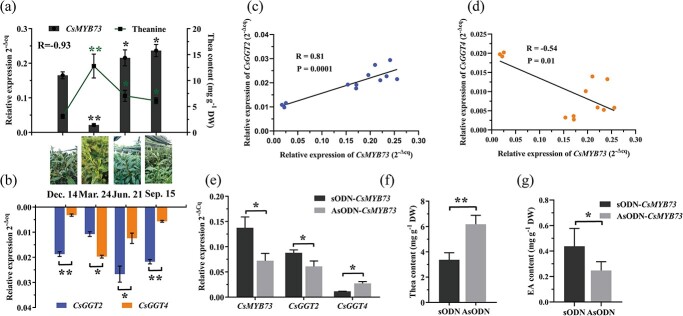
Correlation analysis of theanine accumulation in tea shoots from different seasons. **(a)** Analysis of the correlation between theanine content and *CsMYB73* expression. The left Y-axis represents relative *CsMYB73* expression in tea shoots based on various seasons (collected on 14 December 2021, 24 March 2022, 21 June 2022, and 15 September 2022). The right Y-axis represents theanine contents in tea shoots from various seasons, as quantified by HPLC. **(b)** The expression of *CsGGT2* and *CsGGT4* in tea shoots from different seasons. **(c)** The correlation analysis was conducted between *CsMYB73* and *CsGGT2* expression levels. **(d)** The correlation analysis was conducted between *CsMYB73* and *CsGGT4* expression levels*.***€** Expression of *CsMYB73, CsGGT2*, and *CsGGT4* in control leaves (treated with sODN-*CsMYB73*) and *CsMYB73*-silenced leaves (treated with AsODN- *CsMYB73*). **(f)** and **(g)** Theanine and ethylamine (EA) content in control leaves treated with sODN-*CsMYB73* and *CsMYB73*-silenced leaves treated with AsODN-*CsMYB73*.

In tea plant, CsMYB73, a transcription factor specific to R2R3-MYB, negatively regulated theanine biosynthesis. The contents of theanine and the expression of *CsMYB73*, *CsGGT2*, and *CsGGT4* were determined in four different seasons. Based on Fig. 4a and b, we employed the sampling standard of ‘one apical bud with two leaves’. In the spring (24 March), there was the highest concentration of theanine in tea shoots, along with the lowest expression of *CsMYB73*. As the expression levels of *CsMYB73* continued to increase, the content of theanine maintained a downward trend. Therefore, a strong negative correlation was found between *CsMYB73* expression in various tissues and theanine levels (R = −0.93) (Fig. 4a and b). As shown in Fig. 4, a positive correlation was observed between *CsMYB73* and *CsGGT2* (R = 0.81) (Fig. 4c), while a negative correlation was observed with *CsGGT4* (R = −0.54) (Fig. 4d). The suppression of *CsMYB73* resulted in the repression of *CsGGT2* expression, while *CsGGT4* was activated (Fig. 4e). Further analysis revealed that theanine content had been sharply reduced, along with ethylamine content with a significant increase in tea leaves (Fig. 4f and g).

### *CsMYB73* targets and differentially regulates the promoters of *CsGGT2* and *CsGGT4*

In general, MYBRs present in target gene promoters are bound by R2R3-MYB transcription factors (TFs) [30]. In this study, we cloned the promoters of *CsGGT2* (*pro-CsGGT2*) and *CsGGT4* (*pro-CsGGT4*) and found one specific MYB element in *pro-CsGGT2* and *pro-CsGGT4* sequences (Fig. S3a and b). As shown in Fig. S3c, subcellular localization analysis confirmed CsMYB73 as an R2R3-MYB transcription factor located in the nucleus (Fig. S3d).

**Figure 5:**
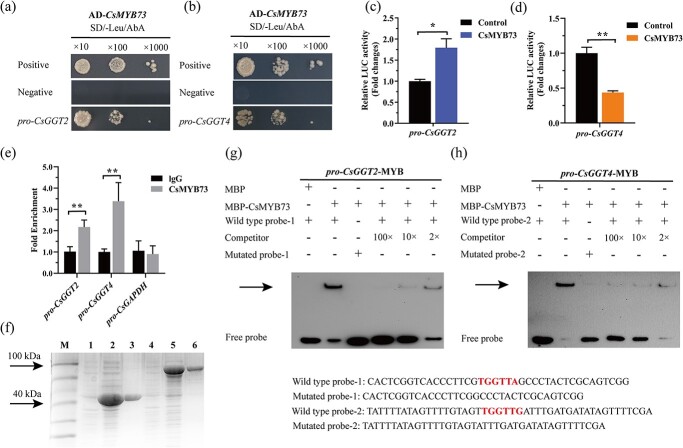
CsMYB73 differentially regulates the expression of *CsGGT2* and *CsGGT4*. **(a)** and **(b)** Y1H assay revealed CsMYB73 binds to both *CsGGT2* and *CsGGT4* promoters. Positive controls for the assays included pGADT7–53 and p53-pAbAi, whereas pGADT7-T and p53-pAbAi were used as negative controls. The concentrations of AbA utilized were 200 μg/L and 400 μg/L. **(c)** and **(d)** Luciferase reporter assays were used to assess the impact of CsMYB73 on the activity of *CsGGT2* and *CsGGT4* promoters. **(e)** A ChlP-qPCR analysis was conducted to determine the binding site of CsMYB73 on the *CsGGT2* and *CsGGT4* promoters. As a mock control, ChlP was incubated with IgG antibody in the current study. The *CsGAPDH* promoter was used as an internal reference for ChIP-qPCR. **(f)** SDS–PAGE analysis of recombinant protein rCsMYB73. Lanes 1, 2, and 3 represent the uninduced, induced and purified pMAL-c5x vector protein, respectively. Lanes 4, 5, and 6 represent the uninduced, induced, and purified recombinant protein rCsMYB73, respectively. **(g)** and **(h)** For EMSA, purified protein rCsMYB73 was combined with various probes, addition is represented by (+), while subtraction is represented by (−). In order to evaluate the binding abilities of the rCsMYB73 protein, different concentration ratios between the cold probe and the biotin probe were used. The labeled probe and mutated probe sequences are provided here, with red highlighting for protein binding sites. Assays were carried out on three independently conducted experiments and data were presented as means with standard deviations (*n* = 3). Student’s *t* test indicates statistical significance by asterisks (^*^P < 0.05, ^**^P < 0.01).

To understand how *CsGGT2* and *CsGGT4* were transcriptionally regulated, we explored the potential for CsMYB73 to physically interact with the promoters of CsGGT2 and CsGGT4 by yeast-one-hybrid (Y1H). As illustrated in Fig. 5a and b, no basal activities of *pro-CsGGT2* and *pro-CsGGT4* were detected in yeast exposed to Aureobasidin A (AbA). While coexpressed with CsMYB73, AbA was driven by *CsGGT2* and *CsGGT4* promoters. Therefore, it can be inferred that the growth of yeast cells was robust when exposed to AbA, suggesting that CsMYB73 possesses the ability to interact with *pro-CsGGT2* (Fig. 5a) and *pro-CsGGT4* (Fig. 5b). The dual luciferase assay was performed to further investigate the regulatory of *CsGGT2* and *CsGGT4* by CsMYB73 in *Nicotiana benthamiana* leaves. As demonstrated in Fig. 5c and d, the activity of *pro-CsGGT2* was obviously promoted in the presence of CsMYB73, while the activity of *pro-CsGGT4* was obviously repressed by CsMYB73 compared to control plants transformed with empty pGreen II 0800. A ChlP-PCR analysis confirmed that CsMYB73 binds to the promoters of *CsGGT2* and *CsGGT4* in tea plants (Fig. 5e). To determine whether CsMYB73 directly binds to the promoters of *CsGGT2* and *CsGGT4*, recombinant protein MBP-CsMYB73 was purified, and biotin probes containing MYB-binding sites on *pro-CsGGT2* and *pro-CsGGT4* were synthesized for EMSAs (Fig. 5f). EMSAs demonstrated that the MBP protein exhibited an inability to bind to the *pro-CsGGT2* and *pro-CsGGT4* sequences, whereas MBP-CsMYB73 displayed specific binding to *pro-CsGGT2* and *pro-CsGGT4*, which was diminished when unlabeled cold probes or mutant probes were employed (Fig. 5g and h).

### Inhibition of *CsMYB73* increased theanine content mediated by *CsGGT2* and *CsGGT4* in the shoots of ‘Huangkui’ during the yellowing stage

**Figure 6:**
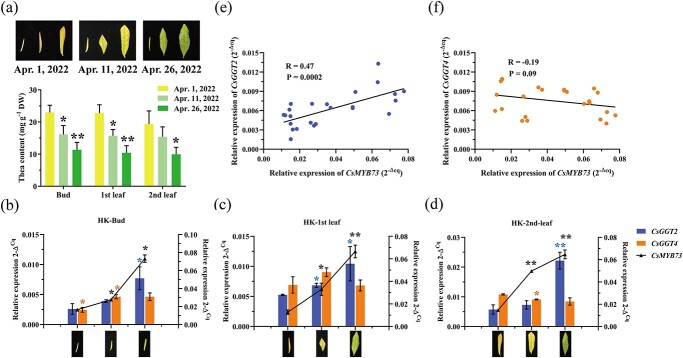
Gene expression and theanine accumulation in tea shoots of ‘Huangkui’ during the process returning green from yellow. **(a)** The bud, first leaf, and second leaf of ‘Huangkui’ collected on three different dates (1 April, 11 April, and 26 April 2022). Theanine accumulation levels in various tissues of ‘Huangkui’ collected on three different dates. **(b)** Expression of *CsGGT2*, *CsGGT4*, and *CsMYB73* in the bud of ‘Huangkui’ collected on three different dates. **(c)** Expression of *CsGGT2*, *CsGGT4*, and *CsMYB73* in the first leaf of ‘Huangkui’ collected on three different dates. **(d)** Expression of *CsGGT2*, *CsGGT4*, and *CsMYB73* in the second leaf of ‘Huangkui’ collected on three different dates. **(e)** The correlation analysis was conducted between *CsMYB73* and *CsGGT2* expression levels in various tissues of ‘Huangkui’ collected on three different dates. **(f)** The correlation analysis was conducted between *CsMYB73* and *CsGGT4* expression levels in various tissues of ‘Huangkui’ collected on three different dates*.* Assays were carried out on three independently conducted experiments and data were presented as means with standard deviations (*n* = 3). Student’s *t* test indicates statistical significance by asterisks (^*^P < 0.05, ^**^P < 0.01).

For further investigation of CsMYB73’s regulatory impact on *CsGGT2* and *CsGGT4*, samples of new shoots (bud, first leaf and second leaf) of ‘Huangkui’ were collected on three different dates (1 April, 11 April, and 26 Apr 2022), during the process of yellowing to green turning (Fig. 6a). The theanine content in different leaf positions was found to be highest in the yellowing shoots and decreased as the shoots returned to green (Fig. 6a). Notably, the expression of *CsMYB73* was observed to be at the lowest level on 1 April and significantly increased as the shoots transitioned from yellowing to green (Fig. 6b–d). Additionally, the expression levels of *CsGGT2* also continued to increase during shoot green turning, while the expression of *CsGGT4* slightly decreased (Fig. 6c and d). Hence, CsMYB73 contributes to regulate the expression of *CsGGT2* and *CsGGT4*, thereby influencing theanine accumulation in the leaves of ‘Huangkui’ during the transition from yellowing to greening. The correlation analysis results indicate a positive correlation between *CsMYB73* and *CsGGT2* (R = 0.47) (Fig. 6e), while a negative correlation was observed between *CsMYB73* and *CsGGT4* (R = −0.19) (Fig. 6f). Notably, a significant decrease in *CsMYB73* expression was observed in yellowing leaves of ‘Huangkui’, during the yellowing period. The inhibition of theanine hydrolase CsGGT2 activity, and the activation of theanine synthase CsGGT4 activity, resulted in a higher level of theanine accumulated in tea shoots.

## Discussion

### The differential protein structure between CsGGT2 and CsGGT4 implied their different biological functions in tea plants

In our previous study, CsGGT2 was confirmed to act as a theanine hydrolase and was regulated by light in tea leaves [19]. Two GGT proteins, CsGGT2 and CsGGT4, have been identified in tea plants [18], and this study continued to explore the functional differences and regulatory mechanisms between them. It has been demonstrated in the present study that recombinant proteins rCsGGT2 and rCsGGT4 play dual roles in both theanine synthesis and degradation *in vitro* and in plant. However, they showed quite different catalytic efficiencies for theanine synthesis and degradation (Fig. 1). This phenomenon may have been caused by the structural difference between CsGGT2 and CsGGT4 proteins [31].

The kinetic analysis showed that the difference in affinity for different substrates was slight, which may have been caused by the structures of the recombinant proteins not completely replicating the models in tea plants. It is known that most GGT proteins contain a lid loop that covers the glutamate binding site. According to previous studies, the lid loops have been observed to act as gating structures, which allows the small glutamine to be selected over other substrates [31]. Therefore, the catalytic efficiency of rCsGGT2 and rCsGGT4 toward glutamine was much higher than that toward theanine (Table 1). Therefore, it is necessary to continue to deeply explore the protein structures of CsGGT2 and CsGGT4 in a follow-up study.

Homology modeling and structural analysis showed that CsGGT2 and CsGGT4 proteins have similar structures, and the 2D and 3D structures of CsGGT4 exhibits more compact and complex folding poses, which affects the binding efficiency of proteins with small molecules (Fig. S2, see online supplementary material). As reflected in this study, theanine preferentially binds to the CsGGT2 protein, while EA preferentially binds to the CsGGT4 protein (Fig. 3a and c). These results further reflected that the different protein structures could influence protein function by affecting the binding efficiency between proteins and substrate molecules.

### Theanine dynamic accumulation in tea plants: a complex process

Theanine, an important amino acid in tea plants, is present in tea leaves in the highest concentration among all free amino acids. The processes of synthesis and degradation of theanine are recognized as crucial mechanisms for maintaining a dynamic equilibrium between nitrogen storage and recycling in tea plants [1, 32]. Previous studies have indicated that the accumulation of theanine is subject to dynamic regulation influenced by various factors, such as the growth of tea plants and environmental factors, including development, season, temperature, light, and other abiotic and biotic stresses [33–35]. Tea plants accumulate theanine dynamically as a result of biosynthesis, degradation, and transport processes, all of which are coregulated by genetics, nitrogen status, and the environment [1, 6, 36].

The genes encoding proteins involved in theanine synthesis and degradation are not the only ones in tea plants. Previous reports have indicated that CsTS1, CsGSs, and CsGGTs are enzymes that facilitate theanine synthesis from glutamic acid and ethylamine, while CsPDX2.1 exhibits as a theanine hydrolase *in vitro* [17]. Previous studies have shown that light-activated CsGGT2 activity and theanine accumulation in tea leaves are negatively correlated [19]. However, the precise roles and regulatory patterns of CsGGT4 in tea plants have yet to be elucidated.

A new mechanism for regulating theanine accumulation dynamically is described in this study, facilitated by CsMYB73’s regulation of *CsGGT2* and *CsGGT4*, which are members of the same gene family but exhibit preferences for theanine degradation and theanine synthesis, respectively. This study has observed notable variations in the expression patterns of *CsGGT2* and *CsGGT4* in tea shoots at various developmental stages (Fig. 4a). This observation is further supported by the seasonal greening of ‘Huangkui’ shoots (Fig. 6a). Intriguingly, the accumulation level of theanine in tea shoots consistently exhibits a significant negative correlation with the expression of *CsGGT2*, while displaying a positive correlation with the expression of *CsGGT4* (Figs 4e and f and 6e and f). The present study validates the significant contributions of CsGGT2 and CsGGT4 to the degradation and synthesis of theanine, respectively, through *in vitro* enzymatic properties and *in vivo* functional validation analysis (Figs 1–3). Previous research has demonstrated that the expression levels of genes involved in theanine synthesis, transport, and hydrolysis collectively influence the accumulation of theanine in tea shoots [37]. The high accumulation of theanine in Albino or etiolated tea plants can be attributed to the strong biosynthesis and weak catabolism of theanine in new shoots [36]. It is hypothesized that CsGGT2 and CsGGT4 exhibit functional complementarity and collectively contribute to the theanine metabolism process, thereby ensuring the dynamic equilibrium of theanine accumulation in tea shoots. Consequently, conducting a comprehensive exploration of the regulatory mechanisms governing the bifunctional enzymes CsGGT2 and CsGGT4 would yield advantages in terms of tea garden management and the improvement of tea quality.

**Figure 7:**
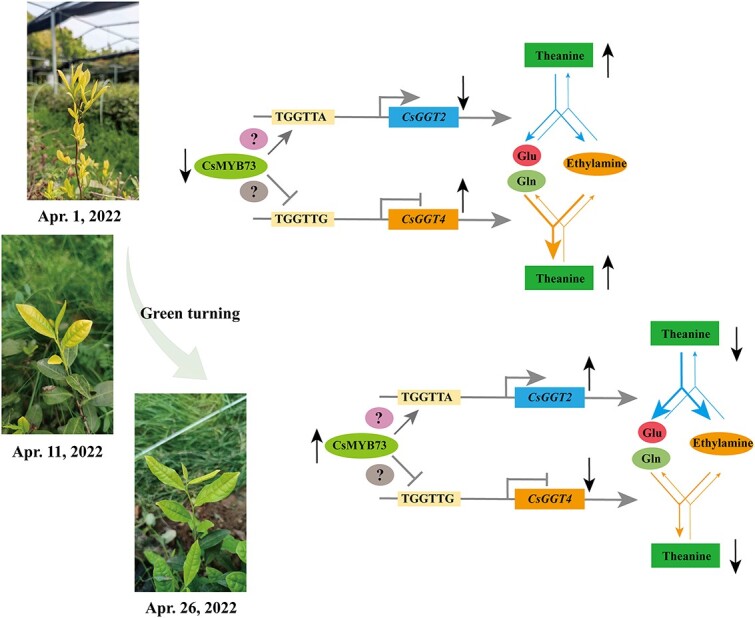
A proposed model for CsMYB73 negatively regulating the accumulation of theanine mediated by *CsGGT2* and *CsGGT4* in tea shoots of ‘Huangkui’ returning green from yellow. The blue arrows indicate theanine metabolism involved in CsGGT2. The orange arrows indicate the theanine metabolism involved in CsGGT4. The black arrows represent upregulated and downregulated gene expression and theanine content. The gray arrows represent a positive effect, and the gray vertical lines represent an inhibitory effect. Glu, glutamine; CsGGT2 and CsGGT4, γ-glutamyl-transpeptidase from tea plants; CsMYB73, transcription factor from tea plants. Ellipses embedded with question marks represent unverified regulatory factors that may participate in this model. During the yellowing period of ‘Huangkui’ leaves, a significant suppression of *CsMYB73* further inhibited the theanine hydrolase CsGGT2 activity, and activated the theanine synthase CsGGT4 activity, resulting in a higher level of theanine accumulated in tea shoots. As tea shoots transition from yellow to green, the upregulation of CsMYB73 leads to an enhancement in the activity of theanine hydrolase CsGGT2, while simultaneously suppressing the synthesis of theanine by the theanine synthase CsGGT4. Consequently, this process culminates in a persistent reduction in the accumulation level of theanine in ‘Huangkui’ shoots.

### CsMYB73 differentially regulates the expression of *CsGGT2* and *CsGGT4* and participates in theanine accumulation in tea shoots

MYB proteins are members of a vast family of the transcription factors found in plant, and they have significant functions in both primary metabolism and secondary metabolism. Increasing evidence suggests that R2R3-MYB transcription factors are involved in regulating the transcription of various genes that encode GS biosynthetic enzymes [38]. In albino tea leaves, CsMYB42 may be implicated in theanine biosynthesis by activating the expression of *CsGS1c* [39]. Additionally, CsMYB73 inhibits theanine synthesis in tea plants by acting as a transcriptional repressor [24]. Based on our analysis, the *CsMYB73* expression levels in tea shoots during various seasons exhibited a consistent pattern with *CsGGT2* expression, indicating an increasing trend as leaf maturation progressed. Conversely, the expression of *CsGGT4* displayed an opposite trend, exerting a negative regulatory effect on theanine accumulation in tea shoots (Fig. 4). It appears *CsMYB73* and *CsGGT2* expression are positively correlated (Fig. 4c), whereas *CsMYB73* and *CsGGT4* expression are negatively correlated in tea shoots across the four seasons (Fig. 4d).

Multiple enzymes are involved in the metabolism of theanine, which is regulated by transcription factors through a precise regulatory mechanism. Multiple studies have documented that R2R3-MYB transcription factor can act as activator and inhibitor, participating in secondary metabolism in tea plants [40]. As well as other transcription factors, these activators and repressors may have specific responses to light, creating a regulatory network that regulates theanine biosynthesis in tea plant, which is worth further exploration [41, 42]. Accordingly, we demonstrate that CsMYB73 exhibits direct binding affinity for *CsGGT2* and *CsGGT4* promoters through Y1H, ChIP-qPCR, EMSA, and dual luciferase assays. This binding activity leads to the activation of *CsGGT2* gene expression, which encodes theanine hydrolase, while simultaneously suppressing the expression of theanine synthase gene *CsGGT4* (Fig. 5). Additionally, the differential regulatory mechanism of CsMYB73 on *CsGGT2* and *CsGGT4* synergistically regulated the accumulation of theanine during the process of ‘Huangkui’ returning to green, which was collected at three time points: 1 April, 11 April, and 26 April (Fig. 6).

Several studies have investigated the mechanisms underlying the increased accumulation of theanine in new shoots of albino/etiolated tea plant cultivars. One aspect of this phenomenon is the correlation between theanine content and the expression levels of genes involved in theanine metabolism [11]. Additionally, the deficiency of chlorophyll may serve as a potential factor contributing to the heightened accumulation of theanine in albino or etiolated tea plants [43]. A previous study has provided evidence of a significant decline in chlorophyll content during the yellowing phase of ‘Huangkui’ shoots, followed by an increase as the leaves transition into green [44]. In this study, there is a continuous decrease in theanine content during the process of ‘Huangkui’ returning to green (Fig. 6a). It is hypothesized that the glutamic acid produced by the degradation of theanine serves as a material basis and energy source for chlorophyll synthesis. Moreover, recent research indicates that the phenotype of albino/etiolated cultivars is also associated with epigenetic changes in tea plants [45]. Consequently, it remains valuable to delve deeper into the mechanisms underlying the seasonal greening of the ‘Huangkui’ cultivar.

The involvement of MBW complexes, comprising R2R3-MYB and bHLH transcription factors, along with WD-repeat proteins, in the regulation of flavonoid biosynthesis is widely recognized within the academic community [42]. Although the ability of CsMYB73 to differentially regulate the expression of *CsGGT2* and *CsGGT4*, as well as its negative influence on theanine accumulation in ten shoots, has been identified, the exact regulatory mechanism remains insufficiently elucidated. As shown in Fig. 7, this study proposes that supplementary transcription factors may interact with CsMYB73, forming protein complexes that play a role in the regulation of theanine metabolism *via* CsGGT2 and CsGGT4 (Fig. 7). Consequently, further research is necessary to ascertain the identity of these transcription factors and unravel the transcription complexes involved in this regulatory network.

## Materials and methods

### Plant material

The two-year-old tea cuttings of ‘Shuchazao’, which were cultivated hydroponically in a greenhouse. The treatments involved either deionized water or 150 mM sodium chloride (NaCl). The nutrient solution composition followed the methodology outlined in previous research [14]. One apical bud and two leaves of ‘Shuchazao’ tea plants from different seasons were collected on 14 December 2021, 24 March 2022, 21 June 2022, and 15 September 2022, respectively. New shoots (the bud, first leaf, and second leaf) of ‘Huangkui’ were collected on 1 April, 11 April, and 26 April 2022. Following collection, the samples were promptly frozen in liquid nitrogen (N_2_) and kept at −80°C until use.

### RNA extraction, cDNA synthesis, and sequence cloning

Total RNA was extracted using the FastPure Cell/Tissue Total RNA Isolation Kit, followed by the synthesized of the first-strand cDNA using the HiScript III 1st Strand cDNA Synthesis Kit (+gDNA wiper). The complete gene sequences were amplified using 2 × Phanta Flash Master Mix (Dye Plus). These kits were all purchased from Vazyme biotechnology company (Nanjing, China). After ligation, the PCR products were cloned into the cloning vector (pEASY-Blunt Zero) and then transformed into competent cells (Trans1-T1 Phage-Resistant), which purchased from TransGen biotechnology company (Beijing, China). Sequencing was used to identify full-length sequences of the amplified products by the General Biotechnology Company (Chuzhou, China).

### Analysis of quantitative real-time PCR

ChamQ Blue Universal SYBR qPCR Master Mix (Vazyme, Nanjing, China) was utilized for conducting the qRT-PCR analyses. The specific primers used in qRT-PCR were listed in Table S1 (see online supplementary material). The data obtained was analysed using the Opticon monitor software (Bio-Rad). As a control, glyceraldehyde-3-phosphate dehydrogenase *CsGAPDH* (Accession no. GE651107) was served as the baseline (0). To ensure accuracy, cDNA synthesis was performed using a minimum of three independent RNA extractions as technical replicates. The results were then analysed using the 2^-ΔCq^ method [46].

### Heterologous expression and enzymatic activity assay

The pMAL-c5x vector was used to clone and insert the complete open reading frame (ORF) of *CsGGT4*, which harbors a maltose-binding protein (MBP) tag with a molecular weight of 42.5 kDa. The protein fused with MBP and was purified using maltose-binding resin [18]. By using a photometric method, protein concentration was determined, and the accuracy of protein sizes was confirmed through the sodium dodecyl sulfate–polyacrylamide gel electrophoresis (SDS–PAGE). The recombinant protein was analysed according to a previously published protocol for enzyme activity [19].

### The analysis of enzyme kinetics and surface plasmon resonance (SPR)

In order to assess the affinity of the recombinant protein rCsGGT4 towards its substrate, the enzyme kinetics and surface plasmon resonance (SPR) analysis were conducted. The kinetic parameters for theanine were determined at pH 8.0, 40°C and reaction for 24 hours, while glutamine and ethylamine hydrochloride were examined at pH 10.0, 30°C and reaction for the same duration in Tris–HCl buffer. The enzymatic reaction system for theanine synthesis and degradation were previously described and analysed using GC–MS and HPLC–MS [19].

A Biacore T200 system was used with some modifications to the manufacturer’s instructions for SPR analysis. Acetate buffers were used with various pH values (pH 4.0, 4.5, 5.0, and 5.5) to dilute the purified MBP protein or rCsGGT4 protein. The experiments were performed according to the previous study [19].

### Homology modeling and molecular docking

In this study, the homology model of CsGGT2 and CsGGT4 was constructed by using γ-glutamyl transpeptidase from *E. coli* as the template (Protein Data Bank ID: 2E0W) [47]. Then, the 3D structures of target proteins and optimization of models were determined by I-TASSER (https://zhanglab.ccmb.med.umich.edu/I-TASSER/) [48].

AutoDock is a collection of automated docking tools specifically to forecast how small molecules bind to receptor proteins with known three-dimensional (3D) structures. To ascertain the potential conformation of both the ligand molecule and the receptor proteins, the Lamarckian genetic algorithm was employed using AutoDock Tools (version 1.5.6) [49]. The structures of glutamine, theanine, and ethylamine were acquired from the PubChem website (https://pubchem.ncbi.nlm.nih.gov/) and were initially subjected to energy and geometry optimization prior to the docking process [50]. Next, receptor proteins were modified with polar hydrogen atoms and Kolhman charges using AutoDock Tools. Similarly, ligand structures were prepared by incorporating polar hydrogen atoms and Gasteiger partial charges, while allowing torsions to rotate [51]. The resulting docked poses were subsequently visualized employing PyMOL software [52]. The docking experiment utilized a population size of 60, and for subsequent analysis, the conformation with the lowest binding free energy was selected [53].

### Transient expression of *CsGGT4* in *N. benthamiana*

For transient expression of *CsGGT4*, we successfully constructed the ORF of the *CsGGT4* into the pK7WGF2 vector and transformed it into *Agrobacterium tumefaciens* strain GV3101 (pSoup–p19) (Weidi). In this study, the empty vector pK7WGF2.0 was used as a control experiment. The detailed transient expression process in tobacco leaves and specific detection methods used were referred to our previous study [19]. In this study, the test data points were derived from a combination of three distinct tobacco leaves, with each treatment consisting of three independent data points. A total of nine independent tobacco leaves were utilized.

### Subcellular localization analysis

To investigate the subcellular distribution of CsMYB73 (Accession number: TEA014193.1), the coding sequence of *CsMYB73* was integrated into a pK7WGF2.0 vector, which contained a green fluorescent protein (GFP). The resulting constructs, *CsMYB73*-pK7WGF2.0 and the control pK7WGF2.0 empty vector, were then introduced into *Agrobacterium* and subsequently injected into tobacco leaves for the purpose of transient expression. After a brief period of 2–3 days, fluorescence images were captured using laser scanning confocal microscopy (LSCM) [19].

### Y1H and dual-luciferase assay

The yeast one-hybrid (Y1H) and dual-luciferase experiments were performed according to the published methods [54]. The promoter fragments of *CsGGT2* and *CsGGT4* were cloned and integrated into the pAbAi vector, while *CsMYB73* was integrated into the pGADT7 vector. The positive controls consisted of pGADT7–53 and p53-pAbAi, whereas pGADT7-T and p53-pAbAi were employed as negative controls.

In the dual-luciferase assay, the pGreen II 0800-SK vector (SK) was utilized to incorporate *CsMYB73*, while the pGreen II 0800-LUC vector (LUC) was employed to insert the promoter fragments of *CsGGT2* and *CsGGT4* [55]. The procedures of vector transformation and tobacco transfection were outlined by Wu *et al.* [56]. The leaf discs infiltrated by *Agrobacterium* were collected after 3 days and subsequently analysed using dual-luciferase assay reagents sourced from Yeasen (Shanghai, China).

### EMSA assay

The interaction between the CsMYB73 protein and the promoters of *CsGGT2* and *CsGGT4* was evaluated by EMSA. After cloning the complete *CsMYB73* sequence into pMAL-c5x vector, it was subsequently purified using the aforementioned procedure. Subsequently, the EMSA assay were conducted following the methodology proposed by Gao *et al.* [57]. A diagram of the probe sequences used in this study can be found in Fig. 5h and i.

### ChIP–qPCR

The ChIP assay was conducted in accordance with the instruction of the EpiQuik™ Plant ChIP Kit (New York, NY, USA). In summary, approximately 2 g of tea leaves were fragmented into small fragments and subsequently cross-linked by 1% formaldehyde solution. This chromatin was subsequently subjected to immunoprecipitation using either CsMYB73-specific antibody or nonimmune IgG antibody, both of which were incubated in the microwells provided in the kit [58, 59].The specificity of the CsMYB73 antibody was detected in the leaves of ‘Shuchazao’ by western blot (Fig. S4a, see online supplementary material). As a mock control, the IgG antibody was incubated and used for ChlP-qPCR in the current study. The *CsGAPDH* promoter without binding sites was used as an internal reference to clarify the accuracy of binding sites and exclude the false positive results of ChIP-qPCR assay. Table S1 (see online supplementary material) details the primers used to analyse the immunoprecipitated DNA fragments after reverse cross-linking.

### Gene suppression of *CsGGT4* and *CsMYB73* in tea leaves

The Soligo software (https://sfold.wadsworth.org/cgi-bin/soligo.pl) was used to design specific sequences of antisense oligonucleotides (AsODNs) according to our previous methods (Table S1, see online supplementary material). The *CsGGT4* and *CsMYB73* genes in tea were silenced by injecting 50 μM AsODN-*CsGGT4* or 50 μM AsODN-*CsMYB73* into the ‘Shuchazao’ leaves until the entire leaf was filled with the solution. A control was administered with sense oligonucleotides (*sODN-CsGGT4 or* 50 μM sODN-*CsMYB73*). Following a 24-hour incubation period, the leaves were sampled and kept at −80°C for further analysis [60]. To further explore the biological function of *CsGGT4* in tea plants, tea shoots were subjected to treatment with either deionized water or a solution containing 150 mM sodium chloride (NaCl). During the treatment, the gene *CsGGT4* was silenced by injecting a solution of 50 μM AsODN-*CsGGT4* into tea leaves, while sODN-*CsGGT4* was utilized as a control. Following various durations of treatment (6, 12, 24, 48, 72, and 96 h), the leaves were individually sampled and promptly frozen at −80°C until further utilization. For gene silencing experiment, each test data point was determined from the mixed samples of three independent tea cutting seedings, and each treatment had three independent data points, that is, nine independent cutting seedings of tea plant.

### Western blot analysis

Protein was extracted from tea leaf samples treated with AsODN-*CsGGT4* or sODN-*CsGGT4*. The quantification of protein concentration was conducted using a Bradford protein assay kit (Beyotime, Shanghai, China). A 12.5% SDS-PAGE gel was employed to separate 20 micrograms of plant protein, followed by transfer onto polyvinylidene difluoride PVDF membranes (Beyotime, Shanghai, China). To assess the expression of target proteins, a chemiluminescence assay was utilized, following previously described methods [19]. The specific antibodies of CsGGT2 and CsGGT4 used in this study were custom-made in Fanpu Biotechnology Co., Ltd (Fanpu, Wuhan, China). The specificity of these antibodies were detected in ‘Shuchazao’ leaves by western blot (Fig. S4b, and c, see online supplementary material). The internal control in this investigation was the β-actin protein obtained from a plant (Sangon, Shanghai, China).

### Determination of theanine and ethylamine

The hot water was employed to extract theanine from various tissues of tea leaves, described by the previous method [33]. To investigate the involvement of CsGGT4 in the production and degradation of theanine, samples were collected and examined utilizing HPLC–MS/MS and GC/MS techniques, following established protocols in a previous study [19]. The instrument parameters and methods employed were in accordance with Dong *et al.* [61].

### Statistical analysis

The trials were conducted in triplicate, and the data acquired in this investigation underwent analysis of variance and were displayed as averages with corresponding standard deviations. The SPSS software version 16.0 was utilized for the analysis of variance and multiple comparisons. Duncan’s multiple range test and Student’s *t*-test were utilized to evaluate notable variances at the significant levels of *P* < 0.05 (*), *P* < 0.01 (**), and *P* < 0.001 (***).

## Acknowledgements

The National Key Research and Development Project (2022YFF1003103 and 2021YFD1601105) provided funding for this study, along with the Science Foundation of Anhui Agricultural University (2021yjs-22). It was a pleasure working with Professor Jianhua Zhu from the School of Life Sciences at Anhui Agricultural University.

## Author contributions

The research was designed and conceptualized by J.S.; funding supported by X. W.; the research was performed and written by M.C.; Y.S., K.F., M.F., J.M., and Y.G. helped perform the research; Q.C., L.L., and Z.Z. revised the manuscript.

## Data availability

Detailed data and figures are included in the article and its supplements.

## Conflict of interest statement

It is declared that the authors do not have any conflicting interests.

## Supplementary material

Supplementary data is available at *Horticulture Research* online.

## Supplementary Material

Web_Material_uhae012

## References

[1] Lin S , Chen Z, Chen T. et al. Theanine metabolism and transport in tea plants (*Camellia sinensis* L.): advances and perspectives. Crit Rev Biotechnol. 2023;43:327–41 35430936 10.1080/07388551.2022.2036692

[2] Ashihara H . Occurrence, biosynthesis and metabolism of theanine (γ-glutamyl-L-ethylamide) in plants: a comprehensive review. Nat Prod Commun. 2015;10:803–10 26058162

[3] Yang Y , Li X, Ratcliffe R. et al. Characterization of ammonium and nitrate uptake and assimilation in roots of tea plants. Russ J Plant Physiol. 2013;60:91–9

[4] Chen Z , Lin S, Li J. et al. Theanine improves salt stress tolerance via modulating redox homeostasis in tea plants (*Camellia sinensis* L.). Front Plant Sci. 2021;12:770398 34721495 10.3389/fpls.2021.770398 PMC8554060

[5] Li H , Teng R, Liu J. et al. Identification and analysis of genes involved in auxin, abscisic acid, gibberellin, and brassinosteroid metabolisms under drought stress in tender shoots of tea plants. DNA Cell Biol. 2019;38:1292–302 31560570 10.1089/dna.2019.4896

[6] Chen T , Lin S, Chen Z. et al. Theanine, a tea-plant-specific non-proteinogenic amino acid, is involved in the regulation of lateral root development in response to nitrogen status. Hort Res. 2023;10:170–80 10.1093/hr/uhac267 PMC9909507 36778187

[7] Huang R , Wang J, Yao M. et al. Quantitative trait loci mapping for free amino acid content using an albino population and SNP markers provides insight into the genetic improvement of tea plants. Hort Res. 2022;9:uhab029 10.1093/hr/uhab029 PMC8788373 35040977

[8] Vuong Q , Bowyer M, Roach P. L-Theanine: properties, synthesis and isolation from tea. J Sci Food Agric. 2011;91:1931–9 21735448 10.1002/jsfa.4373

[9] Liu Z , Li H, Liu J. et al. Integrative transcriptome, proteome, and microRNA analysis reveals the effects of nitrogen sufficiency and deficiency conditions on theanine metabolism in the tea plant (*Camellia sinensis*). Hort Res. 2020;7:1865–77 10.1038/s41438-020-0290-8 PMC7192918 32377356

[10] Zhao J , Li P, Xia T. et al. Exploring plant metabolic genomics: chemical diversity, metabolic complexity in the biosynthesis and transport of specialized metabolites with the tea plant as a model. Crit Rev Biotechnol. 2020;40:667–88 32321331 10.1080/07388551.2020.1752617

[11] Liu Z , Wu Z, Li H. et al. L-Theanine content and related gene expression: novel insights into theanine biosynthesis and hydrolysis among different tea plant (*Camellia sinensis* L.) tissues and cultivars. Front Plant Sci. 2017;8:498 28439281 10.3389/fpls.2017.00498 PMC5383724

[12] Li Z , Yang W, Ahammed G. et al. Developmental changes in carbon and nitrogen metabolism affect tea quality in different leaf position. Plant Physiol Biochem. 2016;106:327–35 27380366 10.1016/j.plaphy.2016.06.027

[13] Ruan J , Ma L, Yang Y. Magnesium nutrition on accumulation and transport of amino acids in tea plants. J Sci Food Agric. 2012;92:1375–83 22083631 10.1002/jsfa.4709

[14] Yang T , Xie Y, Lu X. et al. Shading promoted theanine biosynthesis in the roots and allocation in the shoots of the tea plant (*Camellia sinensis* L.) cultivar Shuchazao. J Agric Food Chem. 2021;69:4795–803 33861578 10.1021/acs.jafc.1c00641

[15] Li F , Li H, Dong C. et al. Theanine transporters are involved in nitrogen deficiency response in tea plant (*Camellia sinensis* L.). Plant Signal Behav. 2020;15:1728109 32067561 10.1080/15592324.2020.1728109 PMC7194376

[16] Wei C , Yang H, Wang S. et al. Draft genome sequence of *Camellia sinensis* var. sinensis provides insights into the evolution of the tea genome and tea quality. Proc Natl Acad Sci. 2018;115:E4151–8 29678829 10.1073/pnas.1719622115 PMC5939082

[17] Fu X , Cheng S, Liao Y. et al. Characterization of L-theanine hydrolase *in vitro* and subcellular distribution of its specific product ethylamine in tea (*Camellia sinensis*). J Agric Food Chem. 2020;68:10842–51 32866009 10.1021/acs.jafc.0c01796

[18] Sun J , Chang M, Li H. et al. Endophytic bacteria as contributors to theanine production in *Camellia sinensis*. J Agric Food Chem. 2019;67:10685–93 31479251 10.1021/acs.jafc.9b03946

[19] Chang M , Ma J, Sun Y. et al. γ-Glutamyl-transpeptidase CsGGT2 functions as light-activated theanine hydrolase in tea plant (*Camellia sinensis* L.). Plant Cell Environ. 2023;46:1596–609 36757089 10.1111/pce.14561

[20] Liu Y , Wang K, Espley R. et al. StMYB44 negatively regulates anthocyanin biosynthesis at high temperatures in tuber flesh of potato. J Exp Bot. 2019;70:3809–24 31020330 10.1093/jxb/erz194 PMC6685667

[21] Qiu Z , Yan S, Xia B. et al. The eggplant transcription factor MYB44 enhances resistance to bacterial wilt by activating the expression of spermidine synthase. J Exp Bot. 2019;70:5343–54 31587071 10.1093/jxb/erz259

[22] Zhang S , Chen Y, He X. et al. Identification of MYB transcription factors regulating theanine biosynthesis in tea plant using omics-based gene coexpression analysis. J Agric Food Chem. 2020;68:918–26 31899636 10.1021/acs.jafc.9b06730

[23] Zhang Y , Li P, She G. et al. Molecular basis of the distinct metabolic features in shoot tips and roots of tea plants (*Camellia sinensis*): characterization of MYB regulator for root theanine synthesis. J Agric Food Chem. 2021;69:3415–29 33719427 10.1021/acs.jafc.0c07572

[24] Wen B , Luo Y, Liu D. et al. The R2R3-MYB transcription factor CsMYB73 negatively regulates L-theanine biosynthesis in tea plants (*Camellia sinensis* L.). Plant Sci. 2020;298:110546 32771159 10.1016/j.plantsci.2020.110546

[25] Ishiye M , Yamashita M, Niwa M. Molecular cloning of the gamma-glutamyltranspeptidase gene from a *Pseudomonas* strain. Biotechnol Prog. 1993;9:323–31 7765305 10.1021/bp00021a012

[26] Rossi M , Bolz C, Revez J. et al. Evidence for conserved function of γ–Glutamyltranspeptidase in *Helicobacter* genus. PLoS One. 2012;7:e30543 22348013 10.1371/journal.pone.0030543 PMC3279353

[27] Yang A , Honig B. An integrated approach to the analysis and modeling of protein sequences and structures III. A comparative study of sequence conservation in protein structural families using multiple structural alignments. J Mol Biol. 2000;301:691–711 10966778 10.1006/jmbi.2000.3975

[28] Rost B . Twilight zone of protein sequence alignments. Protein Eng. 1999;12:85–94 10195279 10.1093/protein/12.2.85

[29] Yin W , Zhao F, He Y. et al. The mechanism of Croci stigma in the treatment of melasma based on network pharmacology and molecular docking. J Cosmet Dermatol. 2023;22:2105–14 36852722 10.1111/jocd.15682

[30] Fan Z , Ba L, Shan W. et al. A banana R2R3-MYB transcription factor MaMYB3 is involved in fruit ripening through modulation of starch degradation by repressing starch degradation-related genes and MabHLH6. Plant J. 2018;96:1191–205 30242914 10.1111/tpj.14099

[31] Calvio C , Romagnuolo F, Vulcano F. et al. Evidences on the role of the lid loop of γ-glutamyltransferases (GGT) in substrate selection. Enzym Microb Technol. 2018;114:55–62 10.1016/j.enzmictec.2018.04.001 29685354

[32] Guo J , Zhu B, Chen Y. et al. Potential ‘accelerator’ and ‘brake’ regulation of theanine biosynthesis in tea plant (*Camellia sinensis*). Hort Res. 2022;9:uhac169 10.1093/hr/uhac169 PMC9614919 36324642

[33] Li F , Dong C, Yang T. et al. Seasonal theanine accumulation and related gene expression in the roots and leaf buds of tea plants (*Camellia sinensis* L.). Front Plant Sci. 2019;10:1397 31749819 10.3389/fpls.2019.01397 PMC6842895

[34] Deng W , Wang S, Chen Q. et al. Effect of salt treatment on theanine biosynthesis in *Camellia sinensis* seedlings. Plant Physiol Biochem. 2012;56:35–40 22579942 10.1016/j.plaphy.2012.04.003

[35] Li X , Wei J, Ahammed G. et al. Brassinosteroids attenuate moderate high temperature-caused decline in tea quality by enhancing theanine biosynthesis in *Camellia sinensis* L. Front Plant Sci. 2018;9:1016 30087682 10.3389/fpls.2018.01016 PMC6066615

[36] Zhu B , Qiao S, Li M. et al. Strong biosynthesis and weak catabolism of theanine in new shoots contribute to the high theanine accumulation in albino/etiolated tea plant (*Camellia sinensis*). Beverage Plant Res. 2023;3:2 doi: 10.48130/BPR-2023-0023.

[37] Huang R , Wang Z, Wen W. et al. Comprehensive dissection of variation and accumulation of free amino acids in tea accessions. Hort Res. 2023;10:uhad263 10.1093/hr/uhad263 PMC10833077 38304331

[38] Gómez-Maldonado J , Avila C, de la Torre F. et al. Functional interactions between a glutamine synthetase promoter and MYB proteins. Plant J. 2004;39:513–26 15272871 10.1111/j.1365-313X.2004.02153.x

[39] Xie N , Huang X, Zhou J. et al. The R2R3-MYB transcription factor CsMYB42 regulates theanine biosynthesis in albino tea leaves. Plant Sci. 2023;336:111850 37648117 10.1016/j.plantsci.2023.111850

[40] Zhao X , Zeng X, Lin N. et al. CsbZIP1-CsMYB12 mediates the production of bitter-tasting flavonols in tea plants (*Camellia sinensis*) through a coordinated activator–repressor network. Hort Res. 2021;8:110 10.1038/s41438-021-00545-8 PMC8087823 33931627

[41] Ma D , Reichelt M, Yoshida K. et al. Two R2R3‐MYBproteins are broad repressors of flavonoid and phenylpropanoid metabolism in poplar. Plant J. 2018;96:949–65 30176084 10.1111/tpj.14081

[42] Wang X , Wu J, Guan M. et al. ArabidopsisMYB4 plays dual roles in flavonoid biosynthesis. Plant J. 2020;101:637–52 31626358 10.1111/tpj.14570

[43] Zhao Y , Wang W, Zhan X. et al. CsCHLI plays an important role in chlorophyll biosynthesis of tea plant (*Camellia sinensis*). Beverage Plant Res. 2023; eoo4. doi: 10.48130/bpr-0023-0037.

[44] Liu X , Cao J, Cheng X. et al. CsRVE1 promotes seasonal greening of albino *Camellia sinensis* cv. Huangkui by activating chlorophyll biosynthesis. Tree Physiol. 2023;43:1432–43 37083709 10.1093/treephys/tpad052

[45] Wei K , Yu S, Quan Q. et al. An integrative analysis of metabolomics, DNA methylation and RNA-Seq data reveals key genes involved in albino tea 'Haishun 2′. Beverage Plant Res. 2022;2: doi: 10.48130/BPR-2022-0002.

[46] Schmittgen T , Zakrajsek B. Effect of experimental treatment on housekeeping gene expression: validation by real-time, quantitative RT-PCR. J Biochem Biophys Methods. 2000;46:69–81 11086195 10.1016/s0165-022x(00)00129-9

[47] Okada T , Suzuki H, Wada K. et al. Crystal structure of the gamma-glutamyltranspeptidase precursor protein from *Escherichia coli*. Structural changes upon autocatalytic processing and implications for the maturation mechanism. J Biol Chem. 2007;282:2433–9 17135273 10.1074/jbc.M607490200

[48] Yang J , Yan R, Roy A. et al. The I-TASSER suite: protein structure and function prediction. Nat Methods. 2015;12:7–8 10.1038/nmeth.3213 PMC4428668 25549265

[49] Patnode K , Demchuk Z, Johnson S. et al. Computational protein–ligand docking and experimental study of bioplastic films from soybean protein, zein, and natural modifiers. ACS Sustain Chem Eng. 2021;9:10740–8

[50] Kim S , Chen J, Cheng T. et al. PubChem in 2021: new data content and improved web interfaces. Nucleic Acids Res. 2021;49:D1388–95 33151290 10.1093/nar/gkaa971 PMC7778930

[51] Mohapatra R , Sarangi A, Azam M. et al. Synthesis, structural investigations, DFT, molecular docking and antifungal studies of transition metal complexes with benzothiazole based Schiff base ligands. J Mol Struct. 2019;1179:65–75

[52] Arroyuelo A , Vila J, Martin O. Azahar: a PyMOL plugin for construction, visualization and analysis of glycan molecules. J Comput Aided Mol Des. 2016;30:619–24 27549814 10.1007/s10822-016-9944-x

[53] Jia J , Gao X, Hao M. et al. Comparison of binding interaction between β-lactoglobulin and three common polyphenols using multi-spectroscopy and modeling methods. Food Chem. 2017;228:143–51 28317707 10.1016/j.foodchem.2017.01.131

[54] Lin N , Liu X, Zhu W. et al. Ambient ultraviolet B signal modulates tea flavor characteristics *via* shifting a metabolic flux in flavonoid biosynthesis. J Agric Food Chem. 2021;69:3401–14 33719437 10.1021/acs.jafc.0c07009

[55] Hellens R , Edwards E, Leyland N. et al. pGreen: a versatile and flexible binary Ti vector for *Agrobacterium*-mediated plant transformation. Plant Mol Biol. 2000;42:819–32 10890530 10.1023/a:1006496308160

[56] Wu W , Wang M, Gong H. et al. High CO2/hypoxia-induced softening of persimmon fruit is modulated by DkERF8/16 and DkNAC9 complexes. J Exp Bot. 2020;71:2690–700 31926021 10.1093/jxb/eraa009 PMC7210769

[57] Gao R , Han T, Xun H. et al. MYB transcription factors GmMYBA2 and GmMYBR function in a feedback loop to control pigmentation of seed coat in soybean. J Exp Bot. 2021;72:4401–18 33825878 10.1093/jxb/erab152

[58] Liu W , Chen G, He M. et al. ABI5 promotes heat stress-induced chlorophyll degradation by modulating the stability of MYB44 in cucumber. Hort Res. 2023;10:uhad089 10.1093/hr/uhad089 PMC10273075 37334179

[59] Xie D , Huang H, Zhou C. et al. HsfA1a confers pollen thermotolerance through upregulating antioxidant capacity, protein repair, and degradation in Solanum lycopersicum L. Hort Res. 2022;9:uhac163 10.1093/hr/uhac163 PMC9531336 36204210

[60] Zhao M , Zhang N, Gao T. et al. Sesquiterpene glucosylation mediated by glucosyltransferase UGT91Q2 is involved in the modulation of cold stress tolerance in tea plants. New Phytol. 2020;226:362–72 31828806 10.1111/nph.16364

[61] Dong C , Li F, Yang T. et al. Theanine transporters identified in tea plants (*Camellia sinensis* L.). Plant J. 2020;101:57–70 31461558 10.1111/tpj.14517

