# Supplementary material for: CsMYB73 negatively regulates theanine accumulation mediated by CsGGT2 and CsGGT4 in tea shoots (Camellia sinensis)
Source: Hortic Res. 2024 Jan 10;11(3):uhae012. doi: 10.1093/hr/uhae012 (PMC10923645; doi:10.1093/hr/uhae012)
Supplement: Web_Material_uhae012 [file web_material_uhae012.zip › Supplementary data.docx]

**Supplementary data**

**Figure S1 SPR analysis of recombinant protein rCsGGT4.** Sensorgrams [resonance units (RU) vs. Time (s)] generated by gradient concentrations of glutamine, ethylamine hydrochloride and theanine for the recombinant protein rCsGGT4 using the SPR aptasensor. **(a)** The data from sensorgrams of glutamine at different concentrations (0-100 mM) for recombinant protein rCsGGT4 were fit to a curve to determine the *K_D_* values of the substrate. **(b)** The data from sensorgrams of ethylamine hydrochloride at different concentrations (0-1000 mM) for recombinant protein rCsGGT4 were fit to a curve to determine the *K_D_* values of the substrate. **(c)** The data from sensorgrams of theanine at different concentrations (0-250 mM) for recombinant protein rCsGGT4 were fit to a curve to determine the *K_D_* values of the substrate.


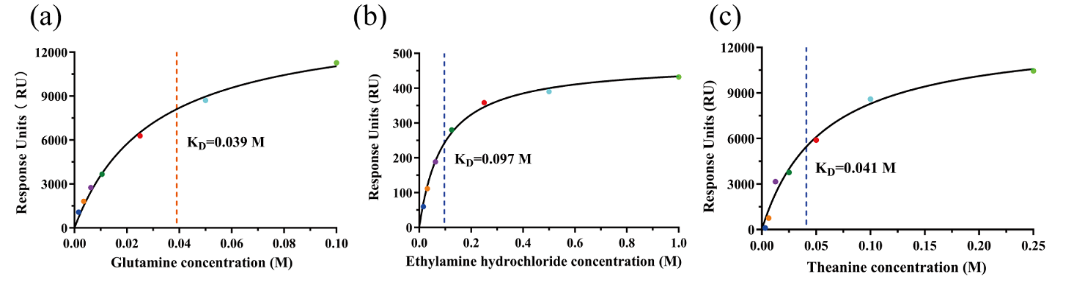


**Figure S2 Sequence alignment and structural analysis of CsGGT2 and CsGGT4.**

**(a)** Comparison of the amino acid sequences of CsGGT2 and CsGGT4 proteins from tea plant. The red boxes mark the catalytic residue threonine (Thr) and nucleophile residue glycine-glycine (Gly-Gly). Other residues responsible for substrate binding and catalytic activity of the proteins are also marked with orange boxes. **(b-g)** The three-dimensional structure of CsGGT2 **(b, d, f)** and CsGGT4 proteins **(c, e, g)**. The black arrow indicates the more compact and complex folding poses of CsGGT4. **(h), (i)** The secondary structure was predicted based on the amino acid sequences of CsGGT2 and CsGGT4. The black boxes indicate the additional helical structures compared to CsGGT2.


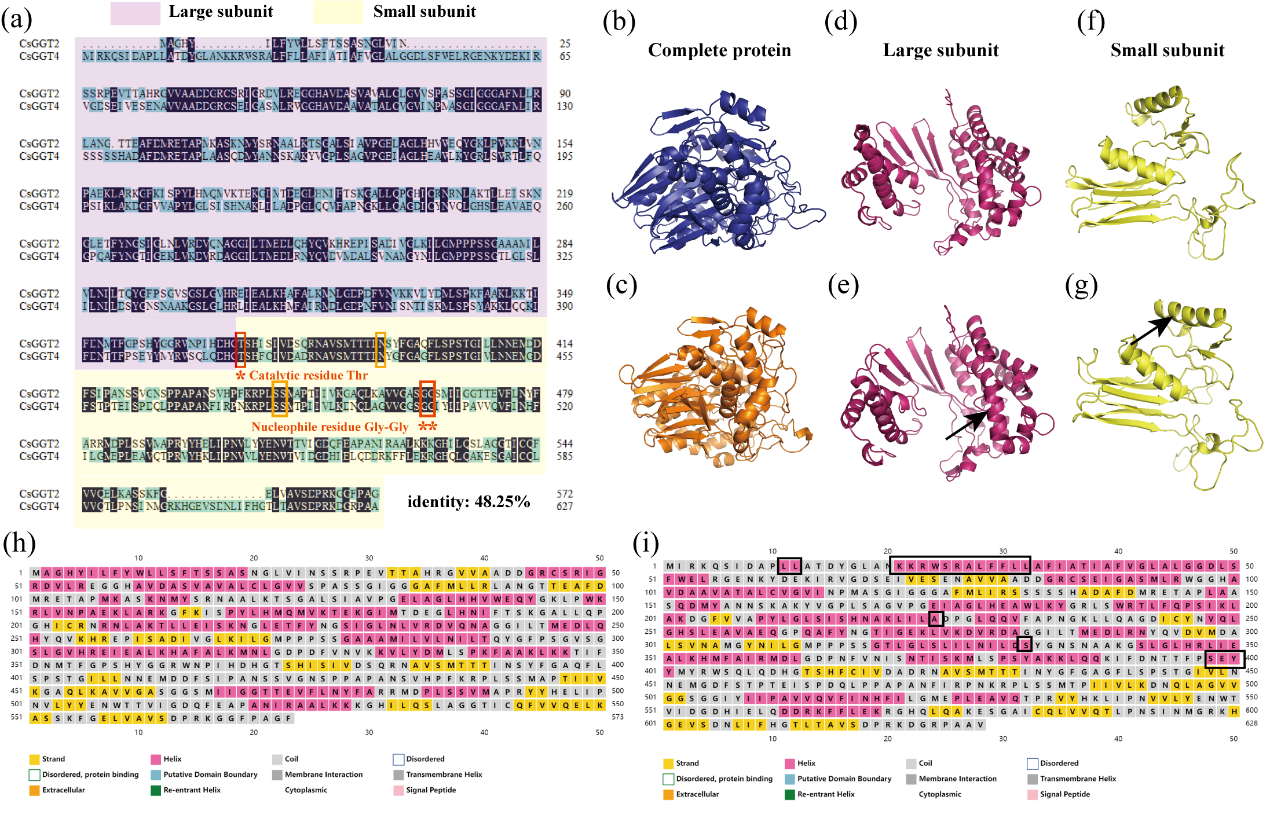


**Figure S3 Sequence analysis and subcellular localization. (a),** **(b)** Mode diagrams of the MYB regulatory elements in the promoters of *CsGGT2* and *CsGGT4* sequences. **(c)** Alignment of the CsMYB73 protein sequence from tea plant with *A. thaliana* AtMYB73 (At4g37260), AtMYB70 (At2g23290) and AtMYB44 (At5g67300) protein sequences. The 22.1 and 22.2 motifs are indicated in black boxes. **(d)** Subcellular localization of the CsMYB73 protein in leaf epidermal cells of *Nicotiana benthamiana*. Bar = 25 µm.


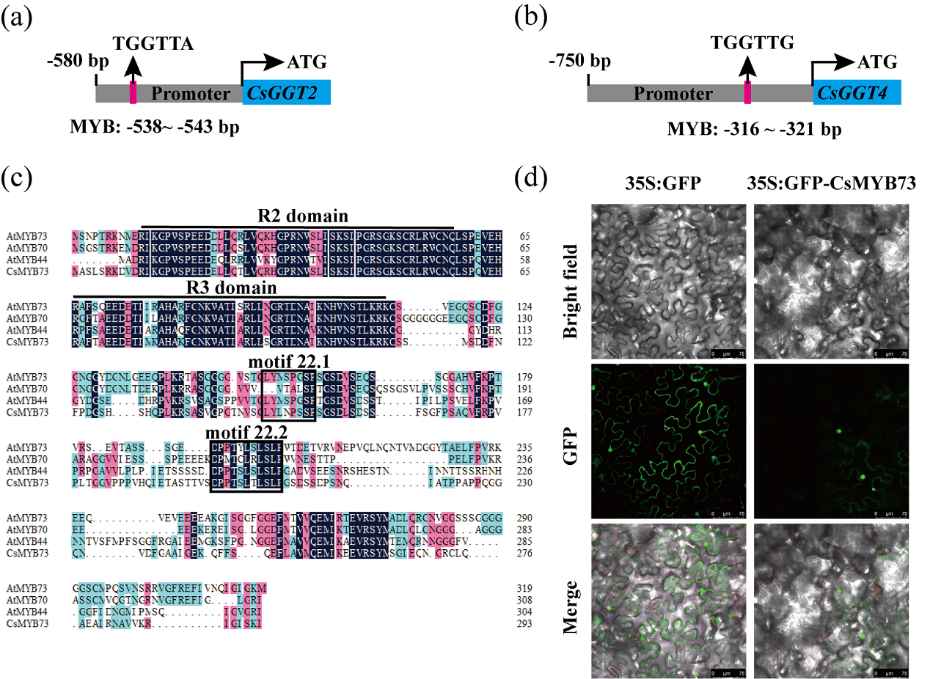


**Figure S4 The specificity of CsGGT2, CsGGT4 and CsMYB73 antibodies in tea leaves by western blot analysis. (a)** Western blot analysis of CsGGT2 protein expression in the leaves of 'Shuchazao' cultivars. **(b)** Western blot analysis of CsGGT4 protein expression in the leaves of 'Shuchazao' cultivars. **(c)** Western blot analysis of CsMYB73 protein expression in the leaves of 'Shuchazao' cultivar. The H_2_O was used as a negative control for western blot. Lanes M represent the molecular mass protein standard, and the black arrows indicate the size of the standard protein.


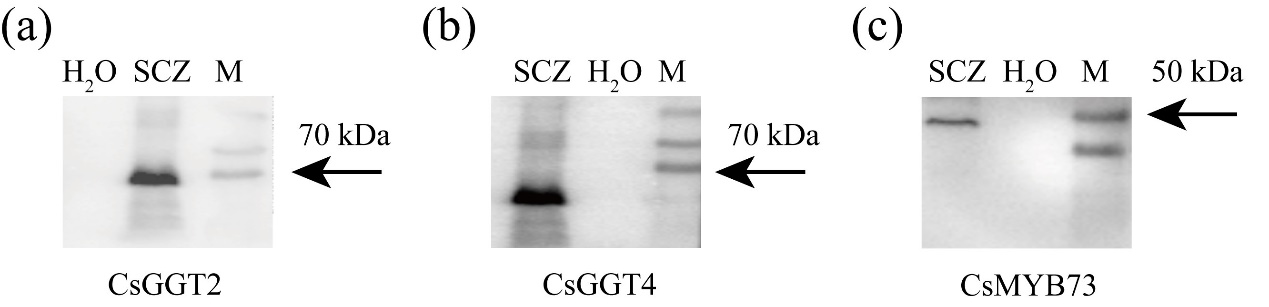


**Table S1** Primer sequences used in this study.

| **Primer name** | **Primer sequence** |
| --- | --- |
| *CsGGT2*-qRT-F | 5'- GAGAAGGAGGTCATGCTGTTGA -3' |
| *CsGGT2*-qRT-R | 5'- AAGCTTTCATGGGTGCAGTTTC -3' |
| *CsGGT4*-qRT-F | 5'- GATGGCTTTGTTGTTGCTCCAT -3' |
| *CsGGT4*-qRT-R | 5'- GAATGCTTGTGGTCCTTGTTCC -3' |
| *CsMYB73*-qRT-F | 5'- ACCTCCCTCACCCTATCCTTAC -3' |
| *CsMYB73*-qRT-R | 5'- CCCGTTTTGTTCAATCCCAGAC -3' |
| *CsGAPDH*-qRT-F | 5'- TTGGCATCGTTGAGGGTCT -3' |
| *CsGAPDH-*qRT-R | 5'- CAGTGGGAACACGGAAAGC -3' |
| sODN-*CsMYB73*-P1 | 5'- CAAGGAACTGGTCGCTGATC -3' |
| sODN-*CsMYB73*-P2 | 5'- GCCCACGCAAAGTTCGGTAA -3' |
| sODN-*CsMYB73*-P3 | 5'- CCGACAACGCGATCAAGAAC -3' |
| AsODN-*CsMYB73*-P1 | 5'- GATCAGCGACCAGTTCCTTG -3' |
| AsODN-*CsMYB73*-P2 | 5'- GATCAGCGACCAGTTCCTTG -3' |
| AsODN-*CsMYB73*-P3 | 5'- GATCAGCGACCAGTTCCTTG -3' |
| sODN-*CsGGT4*-P1 | 5'- CAAGGAACTGGTCGCTGATC -3' |
| sODN-*CsGGT4*-P2 | 5'- GCCCACGCAAAGTTCGGTAA -3' |
| sODN-*CsGGT4*-P3 | 5'- CCGACAACGCGATCAAGAAC -3' |
| AsODN-*CsGGT4*-P1 | 5'- GATCAGCGACCAGTTCCTTG -3' |
| AsODN-*CsGGT4*-P2 | 5'- GATCAGCGACCAGTTCCTTG -3' |
| AsODN-*CsGGT4*-P3 | 5'- GTTCTTGATCGCGTTGTCGG -3' |
| *CsGGT4*-pMAL-F | 5'- CGCGATATCGTCGACGGATCCATGATAAGGAAGCAGAGCATAG -3' |
| *CsGGT4*-pMAL-R | 5'- ACCTGCAGGGAATTCGGATCCTCAGACAGCTGCTGGCCTCC -3' |
| *CsGGT4*-pK7WGF2-F | 5'- GGGGACAAGTTTGTACAAAAAAGCAGGCTTCATGATAAGGAAGCAGAGCA-3' |
| *CsGGT4*-pK7WGF2-R | 5'- GGGGACCACTTTGTACAAGAAAGCTGGGTCTCAGACAGCTGCTGGCCTCC -3' |
| *pro-CsGGT4*-F | 5'- TTTATAAATATACACTGAAATG -3' |
| *pro-CsGGT4*-R | 5'- GTGCTTTAGACGCCCAGTGG -3' |
| *pro-CsGGT4*-pAbAi-F | 5'- AAAATGATGAATTGAAAAGCTTTTTATAAATATACACTGAAATG -3' |
| *pro-CsGGT4*-pAbAi-R | 5'- GAGCACATGCCTCGAGGTCGACGTGCTTTAGACGCCCAGTGG -3' |
| *pro-CsGGT4*-pGreen-F | 5'- GTCGACGGTATCGATAAGCTTTTTATAAATATACACTGAAATG-3' |
| *pro-CsGGT4*-pGreen-R | 5'- GTCGACGGTATCGATAAGCTTGTGCTTTAGACGCCCAGTGG -3' |
| *CsMYB73*-pGADT7-F | 5'- GGAGGCCAGTGAATTCATGGCTTCTTTGTCTAGGAAGGAT -3' |
| *CsMYB73*-pGADT7-R | 5'- CGAGCTCGATGGATCCCTACTCGATCTTGCTAATCCCAATT -3' |
| *CsMYB73*-pGreen-F | 5'- AGCTCCACCGCGGTGGCGGCCGCATGGCTTCTTTGTCTAGGAAGGAT -3' |
| *CsMYB73*-pGreen-R | 5'- GGGGGATCCACTAGTTCTAGACTACTCGATCTTGCTAATCCCAATT -3' |
| *CsMYB73*-pMAL-F | 5'- CCGCGATATCGTCGACATGGCTTCTTTGTCTAGGAAGGAT -3' |
| *CsMYB73*-pMAL-R | 5'- ATTCGGATCCGTCGACCTACTCGATCTTGCTAATCCCAATT -3' |
| *pro-CsGAPDH*-Chlp-F | 5'- GTGGGAAGCTAAGAAGGGCA -3' |
| *pro-CsGAPDH*-Chlp-R | 5'- GCCTGTGGTCGTGTTTTGTG -3' |
| *pro-CsGGT2*-MYB-Chlp-F | 5'- CCCTTCGTGGTTAGCCCTAC -3' |
| *pro-CsGGT2*-MYB-Chlp-R | 5'- GCGGAGGGTGATCAAGTGG -3' |
| *pro-CsGGT4*-MYB-Chlp-F | 5'- TGGGTCCTAGCCATTTTTCAGA -3' |
| *pro-CsGGT4*-MYB-Chlp-R | 5'- GGTGATTGAGGCTGGTTTGTTT -3' |
